# High Duty Cycle to Low Duty Cycle: Echolocation Behaviour of the Hipposiderid Bat *Coelops frithii*

**DOI:** 10.1371/journal.pone.0062938

**Published:** 2013-05-24

**Authors:** Ying-Yi Ho, Yin-Ping Fang, Cheng-Han Chou, Hsi-Chi Cheng, Hsueh-Wen Chang

**Affiliations:** 1 Department of Biological Sciences, National Sun Yat-sen University, Kaohsiung, Taiwan; 2 Department of Biological Resources, National Chiayi University, Chiayi, Taiwan; 3 Department of Leisure and Recreation Studies, Aletheia University, Tainan, Taiwan; 4 Endemic Species Research Institute, Nantou, Taiwan; University of Southern Denmark, Denmark

## Abstract

Laryngeally echolocating bats avoid self-deafening (forward masking) by separating pulse and echo either in time using low duty cycle (LDC) echolocation, or in frequency using high duty cycle (HDC) echolocation. HDC echolocators are specialized to detect fluttering targets in cluttered environments. HDC echolocation is found only in the families Rhinolophidae and Hipposideridae in the Old World and in the New World mormoopid, *Pteronotus parnellii*. Here we report that the hipposiderid *Coelops frithii*, ostensibly an HDC bat, consistently uses an LDC echolocation strategy whether roosting, flying, or approaching a fluttering target rotating at 50 to 80 Hz. We recorded the echolocation calls of free-flying *C. frithii* in the field in various situations, including presenting bats with a mechanical fluttering target. The echolocation calls of *C. frithii* consisted of an initial narrowband component (0.5±0.3 ms, 90.6±2.0 kHz) followed immediately by a frequency modulated (FM) sweep (194 to 113 kHz). This species emitted echolocation calls at duty cycles averaging 7.7±2.8% (*n* = 87 sequences). *Coelops frithii* approached fluttering targets more frequently than did LDC bats (*C.frithii*, approach frequency  = 40.4%, *n* = 80; *Myotis* spp., approach frequency  = 0%, *n* = 13), and at the same frequency as sympatrically feeding HDC species (*Hipposideros armiger*, approach rate  = 53.3%, *n* = 15; *Rhinolophus monoceros*, approach rate  = 56.7%, *n* = 97). We propose that the LDC echolocation strategy used by *C. frithii* is derived from HDC ancestors, that this species adjusts the harmonic contents of its echolocation calls, and that it may use both the narrowband component and the FM sweep of echolocations calls to detect fluttering targets.

## Introduction

Laryngeally echolocating bats listen for echoes of signals to detect prey and obstacles. The diversity of echolocation behaviour reflects the range of ecological situations in which these bats operate [1], [2], [3]. While most (∼700 species) laryngeally echolocating bats separate pulse and echo in time (low duty cycle – LDC), a few (∼180 species) separate pulse and echo in frequency, relying on high duty cycle (HDC) echolocation. They exploit information contained in Doppler-shifted echoes to detect obstacles and locate prey [1], [4]. HDC echolocation is known from species of Hipposideridae and Rhinolophidae in the Old World and the New World mormoopid, *Pteronotus parnellii* [1]. LDC echolocators generally emit calls for less than 25% of the time that they are echolocating, and HDC echolocators for more than 25% of the time [5].

HDC echolocators appear specialized for detecting fluttering targets (flying insects) and use Doppler shift compensation (DSC) to ensure that Doppler-shifted echoes return to the bat within its most sensitive frequency range – the auditory fovea [4], [5]. HDC echolocators are characterized by the presence of auditory foveae, DSC, and narrowband echolocation calls dominated by a single constant frequency. These characteristics provide advantages for echolocation in cluttered environments [5], [6]. Compared to most bat species that use LDC echolocation, HDC echolocators are attracted to and approach fluttering targets more frequently [7]. LDC echolocation prevails in most echolocators (bats, birds, odontocete cetaceans) and is presumed to be the ancestral condition in laryngeally echolocating bats [1], [8], [9], [10]. Until now there has been no evidence of a bat species belonging to HDC taxa (i.e. Hipposideridae, Rhinolophidae and *Pteronotus parnellii*) using LDC echolocation.

Here we report field recordings of free-flying *Coelops frithii*, demonstrating that this hipposiderid uses LDC echolocation, but like HDC hipposiderids, readily detects and approaches fluttering targets. *Coelops frithii* (family Hipposideridae) is a 3.7–6.5 g insectivorous bat that roosts in caves and forages in forest (i.e., acoustically cluttered) habitats. This species is widespread around Southeast Asia, but has received little study [11]. Some reports indicate the absence of a strong narrowband frequency component in its echolocation calls [12], [13].

## Materials and Methods

### Ethics Statement

This study was conducted with the permit issued by the Taiwan Forestry Bureau and with approval from the Institutional Animal Care and Use Committee of National Chiayi University.

### Study area

We conducted field work at a *C. frithii* roost in an abandoned underground shelter in Beinan, Taitung, Taiwan (22°77.1′ N, 121°03.7′ E). From January to April 2011, between 96 and 315 *C. frithii* occupied the roost along with two HDC bat species (11 to 300 *Hipposideros armiger* and 1 to 35 *Rhinolophus monoceros*) and one LDC bat species (0 to 7 individuals of an undescribed *Myotis* species). The roost was located in a broadleaf forest where the bats foraged.

### Echolocation recordings

We recorded echolocation calls of *C. frithii* and the other bats using an Avisoft CM16 condenser microphone and an UltraSoundGate 116 system (Avisoft Bioacoustics, Berlin, Germany) connected to a laptop computer running Recorder v4.1 software. Sounds were digitized at a sampling rate of 1 MHz and resolution of 16 bits, and stored as. wav files.

We recorded calls of free-flying *C. frithii* under various situations, including individuals that (a) passed by and approached the fluttering target, (b) passed by without approaching the fluttering target, (c) passed the roost entrance at dawn or dusk, and (d) were resting inside the roost. We also checked for sexual dimorphism in the echolocation calls by hand-netting and releasing individual male and female *C. frithii* (*n* = 3 per sex) inside the roost, about 2 m in front of the microphone.

### Fluttering target

To test the responses of free-flying bats in the field to fluttering prey, we presented foraging bats with a mechanical fluttering target using a design modified from that described in Lazure & Fenton [7]. We attached a piece of paper (6×17 mm wing area) to a 3 mm diameter plastic tube (7 mm length), cut from the middle section of a cotton tip applicator, and mounted on a metal rod (1.4 mm diameter, 20 cm length). The metal rod was rotated by a battery-powered 6 V DC motor resulting in the paper rotating and fluttering. The plastic tube prevented bats from injuring their flight membranes on the metal rod when attacking the fluttering target. The target could be made to rotate (flutter) at rates between 50 and 80 Hz. Settings within this range were sufficient to induce foraging bats to approach and attack the paper target [7].

We recorded bat calls and filmed bat activity near the fluttering target using a night vision camcorder (PC-350, Sony, Tokyo, Japan) with a wide conversion lens and an infrared light source. The camcorder was positioned 1.5 to 2 m from the fluttering target. Due to the low intensity echolocation signals emitted by *C. frithii*, the recording microphone was placed 0.5 m away from and facing the fluttering target. The microphone was placed either between the target and the camcorder (target-microphone-camcorder) or opposite the camcorder (microphone-target-camcorder), depending on the surrounding vegetation. All three devices were mounted on tripods and elevated ∼0.9 m above ground. We took recordings for between 1 and 3.5 hours immediately after sunset, or 1 to 2 hours before sunrise.

We monitored and recorded behavioural and echolocation responses of LDC and HDC bats within the vicinity of the fluttering target. Following Lazure & Fenton [7], a “bat pass” was defined as a bat passing through the airspace on the video screen with its echolocation calls recorded simultaneously. We defined a bat pass as an “approach” if the bat changed its trajectory to fly towards the target. Otherwise, the pass was classified as “no approach”.

### Sound analysis

We conducted sound analysis on field recordings of *C. frithii*, *H. armiger* and *R. monoceros.* For each individual, we measured the call parameters of 4 to 5 consecutive calls with good signal–to-noise ratio. We used callViewer v18 [14] to measure the following parameters: frequency of maximum energy (FME, kHz), maximum frequency (Fmax, kHz), minimum frequency (Fmin, kHz), pulse duration (d, ms), inter-pulse interval (IPI, ms; defined as time between the termination of one signal and the onset of the successive signal), bandwidth (kHz; defined as Fmax – Fmin), sweep rate (kHz/ms; defined as bandwidth/d), repetition rate (s^−1^; defined as 1/[d + IPI]), and duty cycle (%; defined as d/[d + IPI]*100). Within the *C. frithii* recordings, we also determined the proportion of calls for which the FME was in the second harmonic. We measured only the first harmonic for the LDC species, only the second harmonic for the HDC species, and both harmonics for *C. frithii*. We used SPSS v17 for all statistical analyses. We used multivariate analysis of variance (MANOVA) followed by Tukey's post-hoc tests to determine whether the recording situation, sex, and detection of the fluttering target affected call parameters. We used Pearson's chi-square test to determine if the number of bats approaching the fluttering target differed among species, and applied Bonferroni corrections for subsequent pairwise comparisons. Data are shown as mean ± SD unless otherwise indicated.

## Results

### Echolocation call structure

**Figure 1:**
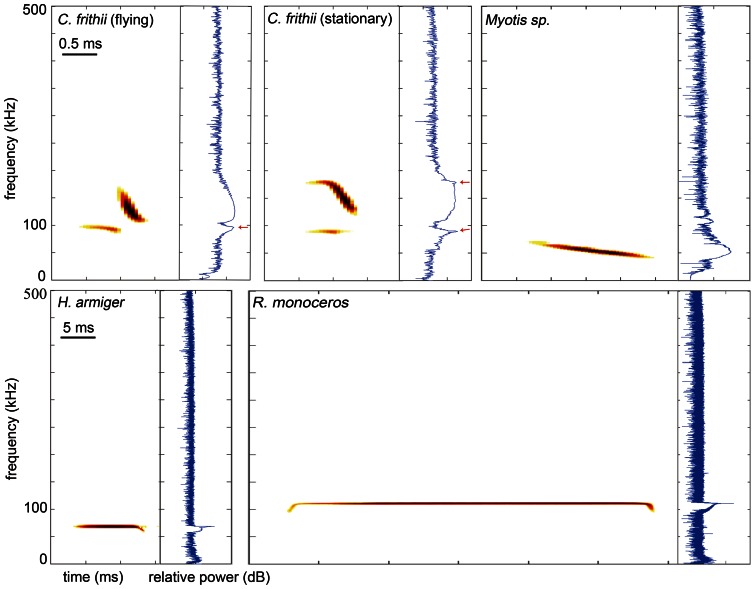
Spectrograms and power spectra illustrating the echolocation calls of ***Coelops frithii*** **,** ***Myotis*** **sp.,** ***Hipposideros armiger*** **, and** ***Rhinolophus monoceros*** **.** Arrows indicate the energy peak of the narrowband components of *C. frithii* calls. Note the time scale is ten-fold different between upper and lower rows. *Short title*: Echolocation call structures of *Coelops frithii*, *Myotis* sp., *Hipposideros armiger*, and *Rhinolophus monoceros*.

**Figure 2:**
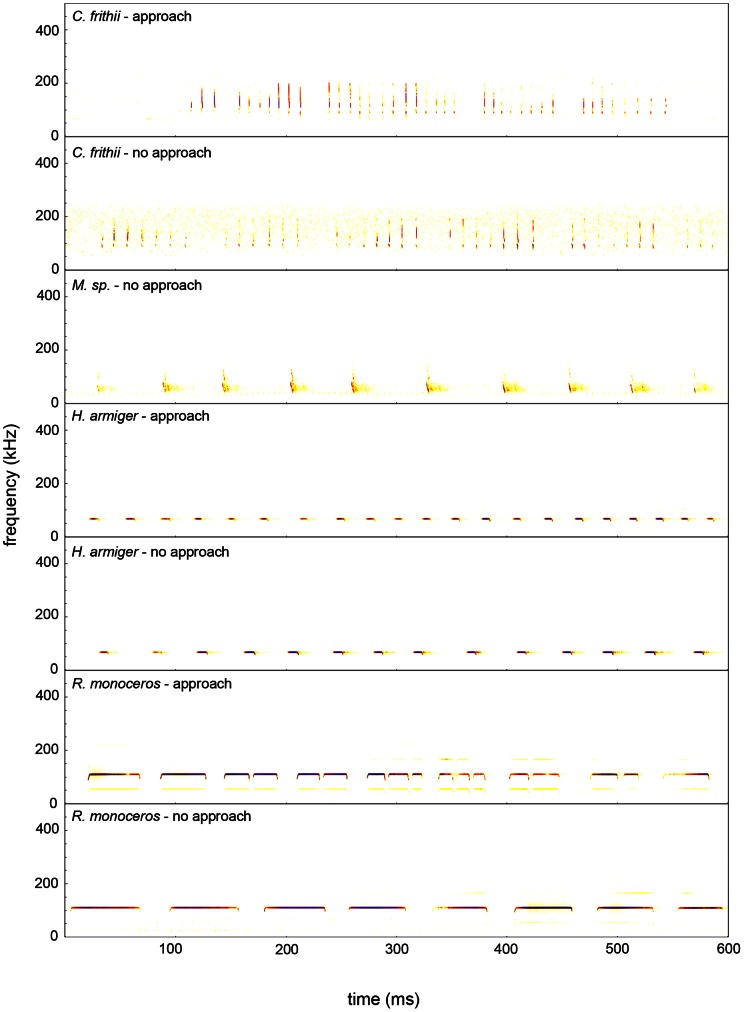
Spectrograms illustrating echolocation call sequences of *Coelops frithii*, *Myotis* sp., *Hipposideros armiger*, and *Rhinolophus monoceros* during passes that both approached and did not approach a fluttering target. *Short title*: Echolocation call sequences of *Coelops frithii*, *Myotis* sp., *Hipposideros armiger*, and *Rhinolophus monoceros*.

We analyzed 87 call sequences (425 individual calls) from *C. frithii* recorded in four situations. Echolocation calls of *C. frithii* are low intensity and were barely detectable when the microphone was within 50 cm of the bat. The calls of *C. frithii* normally consist of a two-part signal: an initial 0.5±0.3 ms narrowband component with an FME in the first harmonic of 90.6±2.0 kHz, followed immediately by an FM sweep ranging from 194 to 113 kHz (Figure 1). Sometimes, the second harmonic of the narrowband component was detected and had an Fmax between 180 and 200 kHz. This was most obvious in the calls emitted by stationary individuals (Figure 1). *Coelops frithii* usually channeled dominant energy into the FM sweep (403 calls) and consistently emitted calls at a high repetition rate (94.6±20.2 Hz) and LDC (7.7±2.8%). Both its echolocation behaviour and call structure differ from other HDC species (Figure 1; Figure 2). Additional details of the calls of *C. frithii* are presented in Table S1.

Signal parameters of *C. frithii* echolocation calls varied significantly among recording situations (1^st^ harmonic, Wilk's λ_24, 168.8_  = 0.411, *P*<0.001; 2^nd^ harmonic, Wilk's λ_24, 221.0_  = 0.341, *P*<0.001; whole call, Wilk's λ_18, 221.1_  = 0.386, *P*<0.001). Overall, calls emitted by stationary bats in the roost were the most distinctive of the four recording situations, differing from all of the flight recordings in at least some parameters (Table S1). We found no sexual dimorphism in the echolocation calls when hand-netting and releasing individual male and female *C. frithii* inside the roost (*n* = 3 per sex; 1^st^ harmonic, Wilk's λ_4, 1_  = 0.151, *P* = 0.554; 2^nd^ harmonic, Wilk's λ_4, 1_  = 0.023, *P* = 0.224; whole call, Wilk's λ_4, 1_  = 0.137, *P* = 0.530; Table S2).

### Flutter detection

We measured the echolocation calls produced during 103 of the 182 bat passes, including those of *C. frithii*, two HDC species (*Hipposideros armiger* and *Rhinolophus monoceros*) and at least two unidentified LDC species (Table 1). Approach rates to the fluttering target differed significantly among species (*χ* ^2^ = 16.43, d.f.  = 3, *P*<0.001; Table 1), with *C. frithii* approaching the fluttering target (40.4% passes) significantly more frequently than other LDC bats (*P* = 0.015), and with the same frequency as the two HDC species (*H. armiger*, *P* = 1; *R. monoceros*, *P* = 0.150).

**Table 1:** Number of bat passes and approaches recorded around the fluttering target.

Species	# of approaches	# of passes	approach rate (%)	duty cycle (%)
*Hipposideros armiger*	8	15	53.3	31.9±5.7 (12)
*Rhinolophus monoceros*	55	97	56.7	66.5±7.3 (51)
*Coelops frithii*	23	57	40.4	6.7±2.7 (31)
low duty cycle bats	0	13	0	6.9±5.2 (9)

Duty cycle data are presented as mean ± SD, with sample sizes shown in parentheses.

When *C. frithii* approached the fluttering target, we recorded significant increases in duty cycle and bandwidth in the second harmonic, accompanied by an increase in pulse duration and maximum frequency. In contrast, both HDC echolocators (*H. armiger* and *R. monoceros*) increased their pulse repetition rates and call duty cycles by reducing pulse duration and inter-pulse interval (Table 2 and Figure 2).

**Table 2:** Comparisons of echolocation call parameters between bats that approached and did not approach to the fluttering target.

	*N*	Duration (ms)	inter pulse interval (ms)	duty cycle (%)	repetition rate (s^−1^)	bandwidth (kHz)	sweep rate (kHz/ms)	Fmin (kHz)	FME (kHz)	Fmax (kHz)
*harmonics combined*										
*Coelops frithii*										
approach	14	1.0	11.5	7.6	82.9	83.9	107.0			
		0.4	1.8	2.6	14.8	11.9	47.6			
no approach	17	0.8	12.0	6.0	80.3	65.6	103.4			
		0.3	1.5	2.6	9.7	16.9	40.3			
F-value		2.141	0.546	3.084	0.343	11.680**	0.053			
*1st harmonic*										
*Coelops frithii*										
approach	12	0.4	12.2	3.6	81.8	7.8	19.8	88.1	90.5	95.9
		0.2	1.8	1.4	13.2	2.6	6.5	2.3	1.6	1.3
no approach	11	0.5	12.3	3.8	80.1	6.7	17.4	89.5	91.0	96.2
		0.2	1.7	2.3	10.6	1.5	6.0	1.8	1.9	2.0
F-value		0.223	0.03	0.119	0.123	1.576	0.887	2.446	0.393	0.109
*2nd harmonic*										
*Coelops frithii*										
approach	14	0.7	11.8	5.4	83.1	61.2	103.2	112.5	132.9	173.7
		0.3	1.9	1.5	14.7	12.8	34.9	4.1	6.6	11.8
no approach	17	0.5	12.2	3.9	80.4	48.7	109.5	115.4	132.4	164.1
		0.1	1.5	0.9	9.9	9.0	32.0	5.3	9.5	11.3
F-value		5.843*	0.501	11.361**	0.382	10.203**	0.279	2.827	0.024	5.318*
*Hipposideros armiger*										
approach	6	6.7	12.1	36.1	58.8			61.7	68.7	69.2
		1.4	4.3	3.6	19.4			0.6	0.5	0.5
no approach	6	9.2	24.8	27.7	29.9			61.3	68.6	68.7
		1.1	4.0	3.9	4.0			1.3	1.0	1.0
F-value		11.838 **	28.781 ***	14.840 **	12.708 **			0.69	0.037	1.185
*Rhinolophus monoceros*										
approach	35	35.8	18.0	68.4	23.9			95.6	109.6	110.1
		10.2	7.0	7.2	13.1			3.2	1.5	1.3
no approach	16	45.7	29.2	62.2	15.3			93.6	108.2	108.7
		10.4	13.2	5.7	6.3			5.4	2.4	2.3
F-value		10.262 **	15.768 ***	9.048 **	6.207 *			2.583	7.157 *	7.571 **

* : *P*<0.05, **: *P*<0.01, ***: *P*<0.001.

(mean on top, SD on bottom).

## Discussion

Our data demonstrate that *C. frithii* produces echolocation calls at a low duty cycle even when detecting and approaching a fluttering target (i.e., hunting for flying insects). Our data contradict previous reports that the echolocation calls of this species lack a narrowband component [12], [13]. We have shown that the structure of the echolocation calls of *C. frithii* differ from those of other HDC bats, which emit calls consisting of a relatively long narrowband pulse with dominant energy and ending in a lower FM sweep.

We propose that *C. frithii* adjusts the harmonic content of its calls and may use either the narrowband component and/or the broadband FM sweep to detect fluttering targets [4], [7], [15], [16]. If *C. frithii* uses DSC and possesses an auditory fovea as do other hipposiderids, it may use the narrowband component for flutter detection. However, due to its LDC value (7.7±2.8%) and extremely short pulse duration [0.5±0.3 ms, less than half of the rotation period of the fluttering target, (6.3–10 ms for rotating frequency at 50–80 Hz)], *C. frithii* is not likely to encode more than one “wingbeat” from the fluttering machine in the echoes from one call. It may not be able to integrate information across a series of calls. Field experiments also indicate that short pulse duration and LDC echolocation are generally associated with a low rate of flutter detection [7]. Therefore, the short narrowband component alone may not explain the high rates of flutter detection by *C. frithii*. We suggest that the FM sweep, the dominant signal component, may also contribute to flutter detection in this species. Although the signal design of LDC echolocation calls does not seem to be specialized for flutter detection, some LDC bat species that use FM echolocation also detect fluttering targets (*Pipistrellus stenopterus* [15]; *Eptesicus fuscus* [17]; species of Murininae and Kerivoulinae [7]). This is probably because the FM Doppler-shifted echoes, produced by the fluttering target from the FM sweep, may be perceived by the bats as two-wavefront echoes with a time separation [15], [16]. Furthermore, when *C. frithii* approaches the fluttering target, increased pulse duration may further increase Doppler-shifted echo delays [16]. While we also recorded an increase in the maximum frequency and bandwidth of calls produced when approaching the fluttering target, we were not able to identify whether these changes were due to the active control of bats or other passive effects. Although *C. frithii* may also use passive hearing to detect prey-generated wingbeat flutter sounds, a strategy adopted by many gleaning bats [2], this would not explain the huge differences in flutter detection performance between *C. frithii* (23 out of 57 passes) and other LDC bats (0 out of 13 passes). The contrast pattern between HDC and LDC echolocators is consistent with the results of previous research [7].

It is likely that the LDC echolocation strategy of *C. frithii* was derived from HDC ancestors. Previous analyses suggest that the HDC approaches in the rhinolophid/hipposiderid lineage optimally only evolved once [1], [8], [18], [19], and morphological and molecular analyses clearly place *C. frithii* among the hipposiderids [20], [21], [22], [23], [24], [25]. Also, the analyses of *FoxP2* and *Prestin* genes, which are likely linked to the function of echolocation in bats, indicate that rhinolophid and hipposiderid bats share a common ancestor [26], [27]. Moreover, although there is no consistent agreement on the phylogenetic relationship between *C. frithii* and other hipposiderids, analyses normally place *C. frithii* in the clade either composed of hipposiderid species known to use HDC echolocation [20], [21], [24] or derived from hipposiderid species that use HDC echolocation [22], [23], [25]. In addition, the analysis of *FoxP2* gene sequences also indicate that *C. frithii* is correctly placed among the rhinolophids and hipposiderids but are more derived than other hipposiderids [26].

Echolocation behaviours of bats are flexible and often influenced more by their ecological situation rather than phylogeny [28]. For example, although *P. parnellii* has also evolved HDC echolocation, it shows no similarities with rhinolophid and hipposiderid bats either in phylogeny or the hearing-related gene, *Prestin* [27]. An intermediate echolocation duty cycle does not occur in the genus *Pteronotus*, and all congeners of *P. parnellii* use only LDC echolocation [29]. Harmonic or frequency shifts have also been documented in sympatric rhinolophids [30] and hipposiderid bats [25]. Compared to rhinolophids, hipposiderid bats emit shorter duration signals and less stable CF resting frequencies. Their auditory foveae are less specialized than those of rhinolophids and they exhibit weaker DSC behaviour [4]. High frequency, broadband FM sweeps may facilitate prey-detection performance by *C. frithii* [3]. Combined with short pulse duration and an LDC strategy, the echolocation behaviour of *C. frithii* may reflect another solution to foraging in acoustically cluttered habitats [2]. Specifically, *C. frithii* appears to benefit by expanding its capacity for prey detection to non-fluttering targets because more than 90% of its diet is spiders (YPF unpublished data).

The use of LDC echolocation by *C. frithii* combined with its ability to detect fluttering targets suggest that HDC echolocation and its associated sophisticated specializations might not be an evolutionary dead end restricting HDC echolocators to cluttered environments [6]. Furthermore, the view that HDC echolocators evolved from an LDC lineage [1], [5] must expand to recognize the possibility of a switch in the opposite direction. Our discovery sets the stage for further work on the evolution of echolocation.

## Supporting Information

Table S1 **Summary of** ***Coelops frithii*** **echolocation call parameters measured in various settings, and results of MANOVA tests.** Approach: bats that approached the fluttering target positioned outside the roost. No approach: bats that did not approach the fluttering target. Entrance: bats flying through the roost entrance at dawn or dusk. Stationary: bats hanging from the ceiling of the roost. (mean on top, and SD on bottom).
(DOC)

Table S2 **Summary of** ***Coelops frithii*** **echolocation call parameters measured from males and females, and results of MANOVA tests between sexes.** (mean on top, and SD on bottom).
(DOC)
