# Supplementary material for: High Duty Cycle to Low Duty Cycle: Echolocation Behaviour of the Hipposiderid Bat Coelops frithii
Source: PLoS One. 2013 May 24;8(5):e62938. doi: 10.1371/journal.pone.0062938 (PMC3663840; doi:10.1371/journal.pone.0062938)
Supplement: Table S1 — Summary of Coelops frithii echolocation call parameters measured in various settings, and results of MANOVA tests. Approach: bats that approached the fluttering target positioned outside the roost. No approach: bats that did not approach the fluttering target. Entrance: bats flying through the roost entrance at dawn or dusk. Stationary: bats hanging from the ceiling of the roost. (mean on top, and SD on bottom). (DOC) [file pone.0062938.s001.doc]

**Table S1.** Summary of *Coelops frithii* echolocation call parameters measured in various settings, and results of MANOVA tests. Approach: bats that approached the fluttering target positioned outside the roost. No approach: bats that did not approach the fluttering target. Entrance: bats flying through the roost entrance at dawn or dusk. Stationary: bats hanging from the ceiling of the roost. (mean on top, and SD on bottom)

| situation | *n* | duration (ms) | inter pulse interval (ms) | duty cycle (%) | repetition rate (s^-1^) | bandwidth (kHz) | sweep rate (kHz/ms) | Fmin (kHz) | FME (kHz) | Fmax (kHz) |
| --- | --- | --- | --- | --- | --- | --- | --- | --- | --- | --- |
| *harmonics combined* | | |  |  |  |  |  |  |  |  |
| approach | 14 | 1.0 | 11.5 a | 7.6 ab | 82.9 a | 83.9 b | 107.0 |  |  |  |
|  |  | 0.4 | 1.8 | 2.6 | 14.8 | 11.9 | 47.6 |  |  |  |
| no approach | 17 | 0.8 | 12.0 a | 6.0 a | 80.3 a | 65.6 a | 103.4 |  |  |  |
|  |  | 0.3 | 1.5 | 2.6 | 9.7 | 16.9 | 40.3 |  |  |  |
| entrance | 28 | 0.8 | 9.7 b | 7.5 ab | 99.6 b | 90.5 b | 134.2 |  |  |  |
|  |  | 0.3 | 2.0 | 2.4 | 20.2 | 14.5 | 51.5 |  |  |  |
| stationary | 28 | 0.9 | 9.3 b | 8.8 b | 104.0 b | 104.8 c | 141.6 |  |  |  |
|  |  | 0.4 | 1.9 | 3.0 | 20.2 | 7.4 | 51.3 |  |  |  |
| F-value |  | 0.973 | 10.589*** | 4.063** | 8.929*** | 34.172*** | 3.141* |  |  |  |
| *1st harmonic* | |  |  |  |  |  |  |  |  |  |
| approach | 12 | 0.4 | 12.2 a | 3.6 | 81.8 a | 7.8 ab | 19.8 | 88.1 ab | 90.5 ab | 95.9 |
|  |  | 0.2 | 1.8 | 1.4 | 13.2 | 2.6 | 6.5 | 2.3 | 1.6 | 1.3 |
| no approach | 11 | 0.5 | 12.3 a | 3.8 | 80.1 a | 6.7 a | 17.4 | 89.5 b | 91.0 ab | 96.2 |
|  |  | 0.2 | 1.7 | 2.3 | 10.6 | 1.5 | 6.0 | 1.8 | 1.9 | 2.0 |
| entrance | 20 | 0.5 | 10.8 ab | 4.2 | 93.7 ab | 7.5 ab | 22.2 | 90.0 b | 91.8 a | 97.5 |
|  |  | 0.3 | 2.5 | 2.0 | 19.2 | 2.0 | 9.9 | 1.9 | 1.4 | 2.2 |
| stationary | 26 | 0.6 | 9.8 b | 5.5 | 103.2 b | 9.8 b | 21.9 | 87.1 a | 89.6 b | 96.9 |
|  |  | 0.3 | 2.5 | 3.2 | 22.3 | 3.9 | 9.9 | 2.6 | 2.2 | 3.3 |
| F-value |  | 0.659 | 4.501** | 2.354 | 5.874** | 4.184** | 0.878 | 7.069*** | 5.286** | 1.169 |
| *2nd harmonic* | |  |  |  |  |  |  |  |  |  |
| approach | 14 | 0.7 a | 11.8 a | 5.4 b | 83.1 a | 61.2 b | 103.2 | 112.5 ab | 132.9 a | 173.7 b |
|  |  | 0.3 | 1.9 | 1.5 | 14.7 | 12.8 | 34.9 | 4.1 | 6.6 | 11.8 |
| no approach | 17 | 0.5 b | 12.2 a | 3.9 a | 80.4 a | 48.7 a | 109.5 | 115.4 a | 132.4 a | 164.1 a |
|  |  | 0.1 | 1.5 | 0.9 | 9.9 | 9.0 | 32.0 | 5.3 | 9.5 | 11.3 |
| entrance | 28 | 0.6 ab | 9.9 b | 6.0 b | 99.5 b | 73.1 c | 130.4 | 113.5 ab | 138.1 ab | 186.7 c |
|  |  | 0.2 | 2.1 | 1.9 | 20.3 | 13.5 | 37.8 | 2.8 | 9.1 | 14.3 |
| stationary | 28 | 0.7 a | 9.4 b | 7.5 c | 103.9 b | 83.3 d | 127.1 | 110.7 b | 140.1 b | 193.9 c |
|  |  | 0.3 | 1.9 | 1.9 | 20.2 | 7.3 | 38.6 | 5.4 | 7.0 | 5.7 |
| F-value |  | 5.514** | 10.721*** | 17.116*** | 8.751*** | 39.617*** | 2.544 | 4.356** | 4.468** | 29.754*** |

*: *P* < 0.05, **: *P* < 0.01, ***: *P* < 0.001.

Different letters indicate differences among groups inferred from Tukey’s post-hoc test.
