# Supplementary material for: High Duty Cycle to Low Duty Cycle: Echolocation Behaviour of the Hipposiderid Bat Coelops frithii
Source: PLoS One. 2013 May 24;8(5):e62938. doi: 10.1371/journal.pone.0062938 (PMC3663840; doi:10.1371/journal.pone.0062938)
Supplement: Table S2 — Summary of Coelops frithii echolocation call parameters measured from males and females, and results of MANOVA tests between sexes. (mean on top, and SD on bottom). (DOC) [file pone.0062938.s002.doc]

**Table S2.** Summary of *Coelops frithii* echolocation call parameters measured from males and females, and results of MANOVA tests between sexes. (mean on top, and SD on bottom)

| sex | *n* | duration (ms) | inter pulse interval (ms) | duty cycle (%) | repetition rate (s-1) | bandwidth (kHz) | sweep rate (kHz/ms) | Fmin (kHz) | FME (kHz) | Fmax (kHz) |
| --- | --- | --- | --- | --- | --- | --- | --- | --- | --- | --- |
| *harmonics combined* | | |  |  |  |  |  |  |  |  |
| male | 3 | 0.7 | 9.6 | 6.9 | 97.9 | 93.6 | 142.0 |  |  |  |
|  |  | 0.2 | 0.6 | 1.3 | 7.2 | 9.0 | 34.0 |  |  |  |
| female | 3 | 0.7 | 11.1 | 6.4 | 85.9 | 95.4 | 144.6 |  |  |  |
|  |  | 0.2 | 1.6 | 2.3 | 11.7 | 11.5 | 43.9 |  |  |  |
| F-value |  | 0.004 | 2.479 | 0.146 | 2.331 | 0.047 | 0.006 |  |  |  |
| *1st harmonic* | |  |  |  |  |  |  |  |  |  |
| male | 3 | 0.5 | 9.8 | 4.8 | 97.7 | 10.6 | 24.8 | 87.9 | 89.2 | 98.4 |
|  |  | 0.3 | 0.5 | 2.6 | 7.0 | 4.4 | 8.2 | 1.6 | 1.5 | 3.1 |
| female | 3 | 0.4 | 11.5 | 3.2 | 85.7 | 6.5 | 21.4 | 87.6 | 88.9 | 94.1 |
|  |  | 0.2 | 1.7 | 2.3 | 11.8 | 1.4 | 9.9 | 2.9 | 4.9 | 4.1 |
| F-value |  | 0.302 | 2.819 | 0.583 | 2.296 | 2.267 | 0.214 | 0.018 | 0.008 | 2.098 |
| *2nd harmonic* | |  |  |  |  |  |  |  |  |  |
| male | 3 | 0.5 | 9.8 | 4.3 | 98.1 | 63.4 | 140.8 | 118.1 | 142.5 | 181.5 |
|  |  | 0.1 | 0.8 | 1.3 | 7.4 | 16.4 | 11.2 | 6.0 | 11.5 | 10.4 |
| female | 3 | 0.5 | 11.3 | 4.6 | 86.2 | 69.5 | 140.3 | 113.5 | 139.1 | 183.1 |
|  |  | 0.1 | 1.4 | 0.2 | 11.4 | 7.3 | 13.8 | 2.2 | 9.6 | 9.4 |
| F-value |  | 0.578 | 2.537 | 0.153 | 2.316 | 0.349 | 0.003 | 1.531 | 0.153 | 0.037 |
